# Vanillin‐Derived Thermally Reprocessable and Chemically Recyclable Schiff‐Base Epoxy Thermosets

**DOI:** 10.1002/gch2.202200234

**Published:** 2023-02-07

**Authors:** Sathiyaraj Subramaniyan, Matteo Bergoglio, Marco Sangermano, Minna Hakkarainen

**Affiliations:** ^1^ KTH Royal Institute of Technology Department of Fibre and Polymer Technology Teknikringen 58 Stockholm 100 44 Sweden; ^2^ KTH Royal Institute of Technology Wallenberg Wood Science Center (WWSC) Teknikringen 58 Stockholm 100 44 Sweden; ^3^ Politecnico di Torino Department of Applied Science and Technology C.so Duca degli Abruzzi 24 Torino 10129 Italy

**Keywords:** biobased, epoxy, recyclable thermosets, Schiff bases, vanillin

## Abstract

The paradigm shift from traditional petroleum‐based non‐recyclable thermosets to biobased repeatedly recyclable materials is required to move toward circular bioeconomy. Here, two mechanically and chemically recyclable extended vanillin‐derived epoxy thermosets are successfully fabricated by introduction of Schiff‐base/imine covalent dynamic bonds. Thermoset 1 (**T1**) is based on linear monomer 1 (**M1**) with two alcohol end groups and one imine bond, while thermoset 2 (**T2**) is based on branched monomer 2 (**M2**) with three alcohol end‐groups and three imine‐groups. Thermosets are obtained by reaction of monomer 1 (**M1**) and monomer 2 (**M2**) with trimethylolpropane triglycidyl ether. The structure of the monomers and thermosets is confirmed by nuclear magnetic resonance and Fourier transform infrared spectroscopic techniques. Both thermosets exhibit good thermal and mechanical properties and they are stable in common organic solvents. Furthermore, they can be thermally reprocessed through compression molding with good recovery of the mechanical properties. Last but not least, the fabricated thermosets can be rapidly and completely chemically recycled to water‐soluble aldehydes and amines by imine hydrolysis at room temperature in 0.1 m HCl solution. This is promising for development of future materials with multiple circularity by different routes.

## Graphical abstract

Future polymer materials, including thermosets, need to be fully circular ideally by several routes to maximize the number of service‐lives. Here promising extended vanillin‐derived Schiff‐base epoxy thermosets (T1 and T2) are designed and their circularity by both mechanical and chemical recycling are demonstrated.

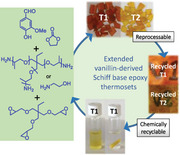

## 1 Introduction

Thermosets are a group of materials with attractive mechanical and thermal properties combined with good solvent resistance.^[^ ^1^, ^2^, ^3^ ^]^ Unfortunately, due to crosslinked molecular structure, traditional thermoset cannot be thermally reprocessed leading to in best case downcycling or incineration of the materials at the end of use phase.^[^ ^4^, ^5^ ^]^ Research focused on development of circular thermosets is, therefore, critical for reaching sustainability goals.

Associative and dissociative covalent adaptable networks^[^ ^6^ ^]^ utilize different dynamic covalent chemistries (DCC) to combine the desired properties of thermosets with the properties typical of thermoplastics, such as thermal reprocessability. Additionally, these materials can often be chemically recycled back to original building blocks under facile conditions compared to traditional polymer materials. Leibler et al. first introduced epoxy vitrimers by using transesterification in 2011.^[^ ^6^ ^]^ After that many DCC reactions have been utilized including transesterification, trans alkylation, transamination, olefin metathesis, Diels‐Alder reactions, disulfide exchange, transcarbamoylation and imine exchanges,^[^ ^7^ ^]^ exploiting, for example, ester,^[^ ^8^, ^9^, ^10^ ^]^ disulfide,^[^ ^11^, ^12^ ^]^ vinylogous urethane,^[^ ^13^, ^14^ ^]^ and imine bonds^[^ ^5^, ^15^, ^16^, ^17^ ^]^

Most synthetic thermosets are produced from fossil‐based resources. Epoxy‐thermosets are at the forefront of biobased thermoset materials, but still >90% of epoxy thermosets are obtained from fossil based resources.^[^ ^18^ ^]^ Epoxy‐thermosets have high performance and enable many products and technologies used in day to day life.^[^ ^19^, ^20^, ^21^, ^22^, ^23^, ^24^ ^]^ However, many epoxy‐thermoset precursors, such as bisphenol A (BPA), are both fossil‐based and toxic and hazardous to the living organisms.^[^ ^18^, ^25^ ^]^ As an example BPA exhibits estrogenic activity and is suspected as human endocrine disruptor.

Schiff base chemistry involving a reaction between aldehyde or ketone with amine has been widely used in biological and medical applications, in catalysis, photo and analytical chemistry. In recent years, it appeared as an attractive DCC for synthesis thermosets containing the imine bond (—C—N—) capable of participating in both dissociative and associative exchange reactions.^[^ ^26^ ^]^ These imine bonds can rearrange at higher temperatures, resulting thermally reprocessable thermosets.^[^ ^27^, ^28^ ^]^ Furthermore, Schiff‐base/imine bond is unstable under acidic conditions enabling chemical recycling under mild conditions.^[^ ^29^ ^]^ Furthermore several aromatic aldehydes, such as vanillin and hydroxymethyl furfural, can be derived from biobased resources and are ideal for production of Schiff‐base thermosets. Many recent papers report photocurable vanillin‐derived Schiff‐base thermosets.^[^ ^5^, ^16^, ^30^ ^]^ Vanillin derived high‐performance Schiff‐base epoxy thermosets with chemical recyclability in acidic medium were also reported.^[^ ^31^ ^]^ Introduction of Schiff‐base could also provide epoxy thermosets with attractive thermal reprocessability.^[^ ^32^, ^33^, ^34^, ^35^, ^36^, ^37^, ^38^ ^]^ Therefore, we developed a facile route to biorenewable‐epoxy thermosets based on extended vanillin monomer recently reported by us.^[^ ^16^ ^]^ The designed thermosets had good thermal and mechanical properties. In addition they were thermally reprocessable and chemically recyclable under acidic conditions at room temperature.

## 2 Result and Discussion

### 2.1 Synthesis of the Monomers and Thermosets

The vanillin‐based imine thermosets were synthesized via three‐step reaction. First the synthesis of monomers involved i) nucleophilic substitution followed by ii) Schiff‐base formation and the synthesis was completed by iii) formation of thermosets. First vanillin **1** was reacted with ethylene carbonate **2** in the presence of potassium carbonate (K_2_CO_3_), under N_2_ atmosphere for 8 h to get the —OH terminated extended vanillin **3**. Then the aldehyde (—CHO) functional group was reacted with 2‐amino ethanol **4** or Jefm **6**, to get monomer 1 (**M1**) and 2 **(M2)**.

**Figure 1:**
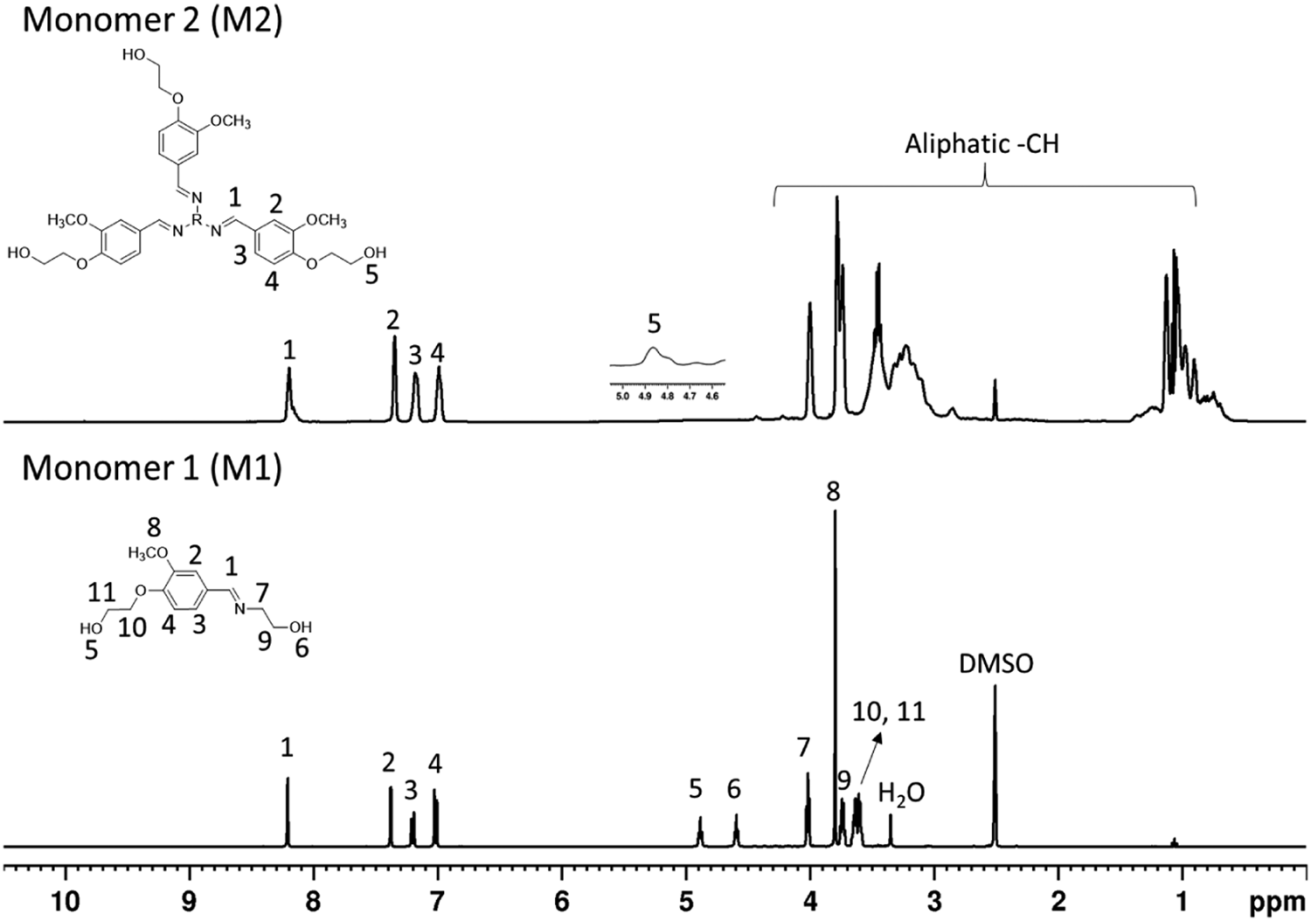
^1^H NMR (400.13 MHz, DMSO‐*d*_6_) spectra of monomer 1 (**M1**) and 2 (**M2**).

**Figure 2:**
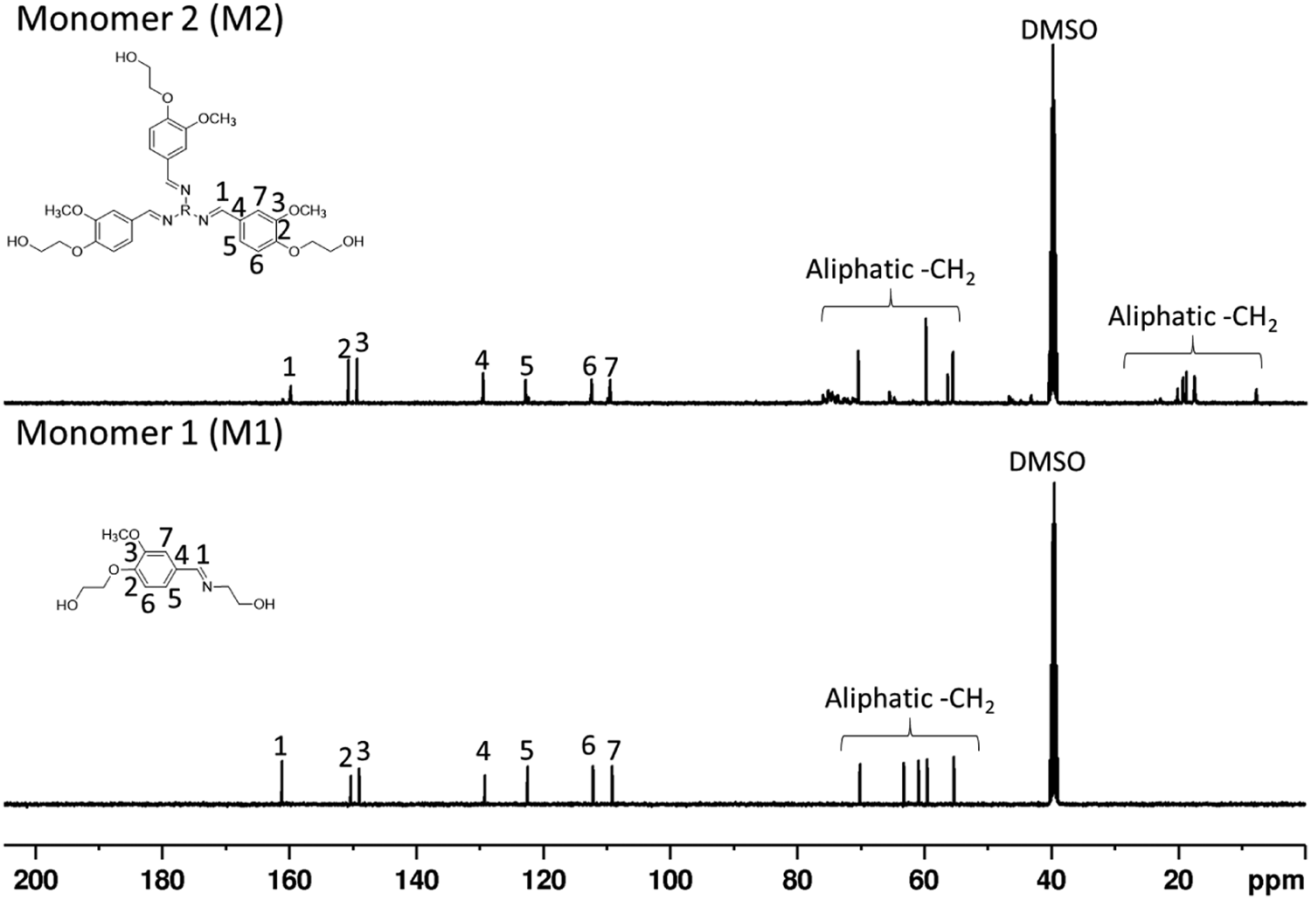
^13^C NMR (100.61 MHz, DMSO‐*d*_6_) spectra of monomer 1 **(M1)** and 2 **(M2)**.

The chemical structure of all the synthesized monomers including the intermediate product **3** (extended vanillin) was confirmed by ^1^H and ^13^C nuclear magnetic resonance (NMR) analysis and the spectra are shown in **Figures** **1** and **2**. For the intermediate product **3**, peaks at δ 4.11 and δ 3.75 ppm correspond to the aliphatic hydrogens, and the peak at δ 4.96 ppm to aliphatic terminal —OH group. The aromatic —OCH_3_, —CH and aldehyde peaks appeared at δ 3.80, 6.50–7.50, and δ 9.80 ppm, respectively. Afterward, the aldehyde group of the intermediate product **3** was involved in the Schiff‐base formation resulting in formation of monomers **5** and **7**, which was unambiguously confirmed by ^1^H and ^13^C NMR spectroscopy. In the ^1^H NMR spectra of all the monomers (Figure 1), the signals of aliphatic —OH group appeared at ≈δ 4.88 and 4.59 ppm, aliphatic —CH_2_ and aromatic —OCH_3_ at ≈δ 1.65 to 4.01 ppm, the imine —CH at δ 8.20 ppm, and the aromatic peaks appeared at ≈δ 6.96 to 7.35 ppm. The structure of the monomers was further confirmed by ^13^C NMR spectroscopy (**Figure** **2**). For all the monomers, the imine carbonyl peak appeared at δ 162.00 ppm and aromatic O—C and aromatic —CH carbon peaks were observed at δ 149.46, 150.87, and δ 129.54 to 109.63 ppm respectively. Aliphatic methylene peaks and aromatic —OCH_3_ peak were observed at δ 70.58 to 7.94 ppm, respectively.

**Figure 3:**
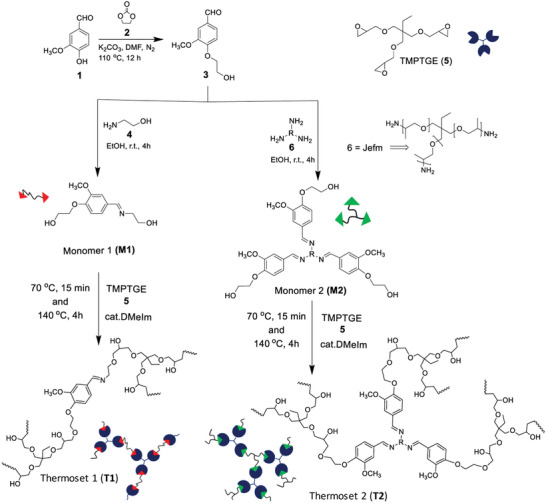
Schematic illustration of the synthesis of Schiff‐base based thermosets 1 (**T1**) and 2 (**T2**).

The curing behavior of the synthesized monomers 1 and 2 **(M1**, **M2)** and trimethylolpropane triglycidyl ether (TMPTGE) was studied by using differential scanning calorimetry (DSC), under non‐isothermal mode (Figures S1 and S2, Supporting Information). The curing reaction was investigated over a wide range of temperature at −30–200 °C and only one exothermic peak was observed. The melting point of monomer 1 (**M1**) was ≈85 °C (Figure S3, Supporting Information). Monomer 2 (**M2**) was a viscous liquid at room temperature and no clear melting point was observed in the studied temperature range. According to the DSC results, a suitable curing temperature for the thermosets 1 (**T1**) and 2 (**T2**) would be at least ≈120 °C and ≈138 °C, respectively. Therefore 140 °C was chosen as the curing temperature for both thermosets. Monomer 1 (**M1)** and monomer 2 (**M2)** were first reacted with TMPTGE (**6**) in the presence of catalytic amount of 1,2‐dimethylimidazole by heating the mixture in a vial at 70 °C for 30 min. Then, while the viscosity of the mixture was still low enough, it was poured into a mold pre‐heated at 140 °C and maintained at this temperature for 3 h to get corresponding cross‐linked thermosets 1 **(T1)** and 2 **(T2)**, see **Figure** **3**.

**Figure 4:**
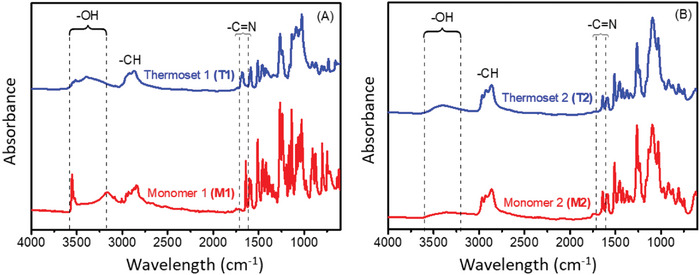
FT‐IR spectra of A) monomer 1 **(M1)** and corresponding thermoset 1 (**T1**), B) monomer 2 **(M2)** and corresponding thermoset 2 **(T2)**.

Furthermore, the monomers and thermosets were further characterized by Fourier transform infrared (FTIR) spectroscopic analysis (**Figure** **4**). For the monomers **(M1** and **M2)** aliphatic and aromatic C—H stretching bands appeared at 2800–3000 cm^−1^, imine peak was observed at ≈1635 cm^−1^, —C—O stretching at ≈1260 cm^−1^ and the aliphatic and aromatic C—H bending bands at ≈750 cm^−1^. To cure thermoset 2 (**T2**), the same method was followed, except that monomer 1 **(M1)** was replaced with monomer 2 **(M2)**.

### 2.2 Solvent Resistance

**Figure 5:**
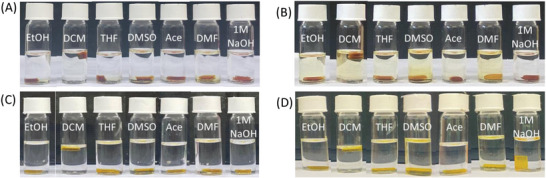
A,C) Initial and B,D) final images of thermoset 1 (**T1**) and 2 (**T2**) after subjection to different solvents at room temperature for 48 h.

The chemical stability of the prepared Schiff‐based thermosets was also investigated by immersing them in different common solvents. Both thermosets remained intact as one piece after immersing them in various solvents (acetone, THF, DMF, DCM, 1 m NaOH, EtOH, DMSO) at room temperature for 48 h. Depending on the solvent, weight losses between 1% and 18% for thermoset 1 **(T1)** and 2–25% for thermoset 2 **(T2)** were observed, as shown in **Figure** **5** and Table S1, Supporting Information.

### 2.3 Thermal and Mechanical Properties

**Figure 6:**
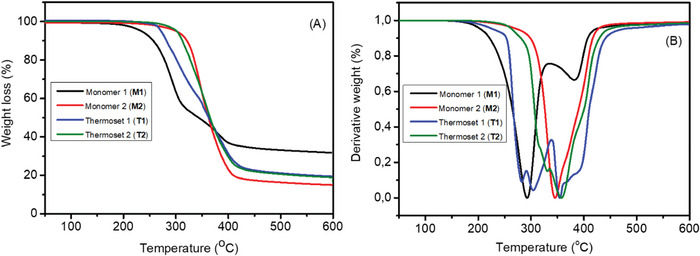
TGA analysis showing the A) weight loss and B) first derivative weight loss curves of monomer 1 **(M1)** and 2 **(M2)** as well as thermoset 1 **(T1)** and 2 **(T2)**.

The thermal stability of the monomers and corresponding thermosets was evaluated by thermogravimetric analysis (TGA) as shown in **Figure** **6** and **Table** **1**. The synthesized monomer 1 (**M1**), thermoset 1 **(T1),** monomer 2 **(M2)** and thermoset 2 **(T2)** had the onset of degradation (*T*_5%_ with 5% wt loss) at ≈238, ≈271, 284, and ≈299 °C, respectively and the maximum degradation temperatures (*T*_d_) were observed at ≈292, 382, and ≈305, 356 °C; ≈344 and ≈354 °C, respectively. Comparison of *T*_5%_ values shows that the thermal stabilities of monomer 1 (**M1**) and thermoset 1 **(T1)** were significantly lower compared to monomer 2 **(M2)** and thermoset 2 **(T2)**. Curing of monomer 1 (**M1**) to thermoset 1 (**T1**), considerably increased the *T*_5%_ temperature, while only a moderate further increase in *T*_5%_ was observed when monomer 2 **(M2)** was cured to thermoset 2 **(T2)**. The reason for this difference in thermal stability is unclear, but it could be connected to the close distance between the imine bond and free hydroxyl‐group in monomer 1 (**M1**) and thermoset 1 (**T1**). The significantly lower thermal stability of monomer 1 (**M1**) compared to monomer 2 **(M2)** could be further influenced by the small molecular size leading to faster formation of volatiles during thermal degradation. The TGA thermograms of thermoset 1 **(T1)** show two degradation steps, while thermoset 2 **(T2)** degrades in one step. Monomer 1 (**M1**) had higher char yield than monomer 2 (**M2**) at 600 °C, which is explained by the higher aromatic content in monomer 1 (**M1**). After crosslinking the expected aromatic content is similar, which was reflected by similar char yields for thermoset 1 (**T1**) and 2 (**T2**) (≈18% and ≈16%, respectively). The thermal transitions of thermosets 1 **(T1)** and 2 **(T2)** were analyzed by DSC. The glass transition temperatures (*T*_g_) of the thermosets 1 (**T1**) and 2 (**T2**) were similar and observed at 32 and 29 °C, respectively, see Figure S4, Supporting Information, and Table 1.

**Table 1:** Thermal properties of thermosets 1 **(T1)** and 2 **(T2)**

Samples	DSC *T*_g_ [°C]	*T*_5%_	*T*_d_	Residue at 600 °C
**T1**	32 ± 1.0	271	282, 305, 356	18%
**T2**	28 ± 0.6	299	354	16%
**M1**	–	238	292, 383	31%
**M2**	–	290	344	13%

### 2.4 Chemical Recyclability

**Figure 7:**
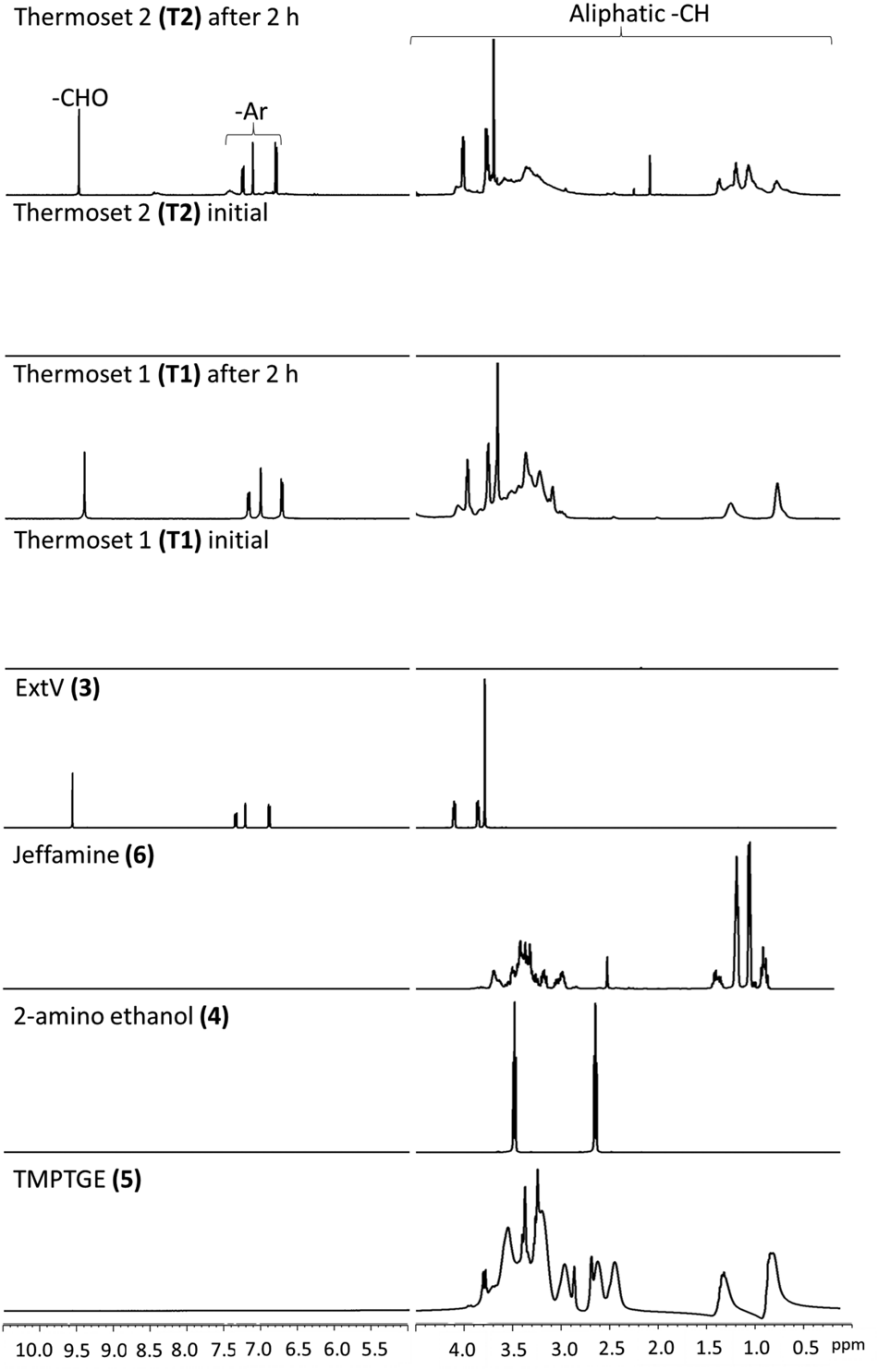
^1^H NMR spectra of A–D) compounds **3**, **4**, **6**, and **8** and E,F) thermoset **1 (T1)** initially and after 2 h and G,H) thermoset **2 (T2)** initially and after 2 h in 0.1 m HCl in D_2_O at room temperature.

**Figure 8:**
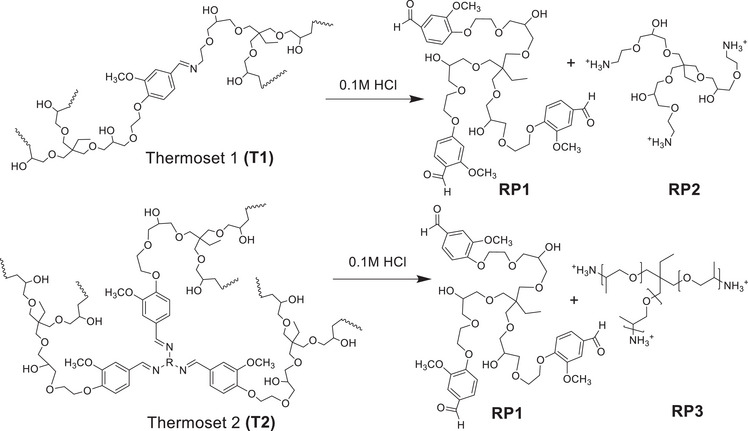
Suggested schematic diagram for the chemical recycling pathway of thermoset 1 **(T1)** and 2 **(T2)** to different recycled products (**RP1**, **RP2**, and **RP3**).

Thermosets are generally resistant to many solvents. This is a benefit during use phase but can complicate recycling. Imine‐bonds in Schiff‐base thermosets, on the other hand, can typically be reversed or opened under aqueous acidic conditions to recover monomeric or oligomeric products that can be recured. We evaluated the possibility to chemically recycle both thermosets by subjecting them to 0.1 m (pH = 1) hydrochloric acid. After immersion of the samples in 0.1 m HCl for 2 h at room temperature, the thermosets were fully dissolved and no solid residues could be visually observed. After 2 h the solvent was evaporated and the residues were dissolved in D_2_O to enable ^1^H NMR analysis. **Figure** **7** shows the ^1^H NMR spectra of water‐soluble products connected to thermoset 1 **(T1)** and thermoset 2 **(T2)** originally and after 2 h of hydrolysis. In addition, the spectra of original building blocks, extended vanillin, TMPTGE, 2‐aminoethanol and Jefm are shown for comparison. No peaks were observed in the ^1^H NMR spectra of thermoset 1 **(T1)** and thermoset 2 **(T2)** at the beginning of the recycling process indicating crosslinked thermoset without significant content of water‐soluble products. After 2 h peaks corresponding to units in the original building blocks were clearly visible, especially those connected to extended vanillin and TMPTGE. The imine hydrolysis was confirmed by appearance of aldehyde (—CHO) peaks at ≈δ 9.40 ppm. The formation and release of water‐soluble vanillin containing products to the acidic water is further supported by the appearance of aromatic —CH peaks at ≈δ 6.90–7.40 ppm. Aliphatic —CH peaks corresponding to the different building blocks are detected at ≈δ 0.5–4.50 ppm. The proposed mechanism, acid hydrolysis of the imine bonds is shown **Figure** **8** and supported by the observed NMR peaks, matching with the expected recycled products RP1, RP2, and RP3 presented in Figure 8.^[^ ^18^, ^26^ ^]^

### 2.5 Mechanical Properties before and after Reprocessing

**Figure 9:**
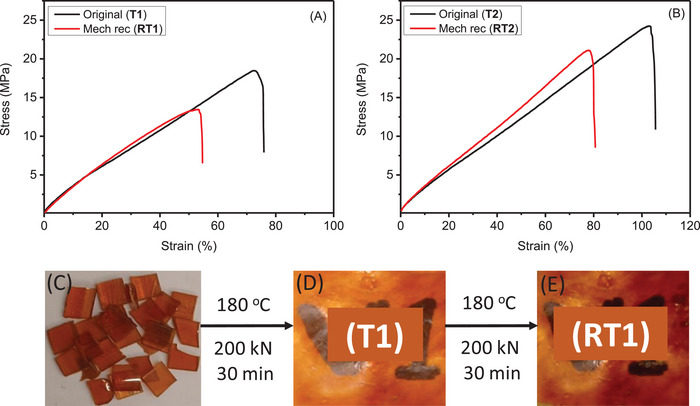
Representative stress–strain curves of A) thermoset 1 **(T1)** and B) thermoset 2 **(T2)** after first compression molding and after mechanical recycling by second compression molding cycle (**RT1** and **RT2**). Digital photos of thermoset 1 (**T1**): C) Cut pieces of oven cured thermosets, D) compression molded original thermoset (**T1**), and E) recycled, second time compression molded thermoset (**RT1**).

The possibility to mechanically recycle the thermosets by reprocessing was evaluated by subjecting the oven cured thermosets to two subsequent compression molding cycles. The mechanical properties after first and second thermal processing were then compared to evaluate the reprocessability. **Figure** **9** shows representative stress–strain curves for thermoset 1 (**T1**) and 2 (**T2**) and the results are further presented in Table S2, Supporting Information. Both thermosets had similar elastic modulus, while thermoset (**T2**) had somewhat higher tensile stress and elongation at break compared to thermoset (**T1**), see Table S2, Supporting Information. The mechanical properties were retained relatively well during the reprocessing with ≈25% reduction in tensile stress at break and elongation at break. Visual images of the materials are also presented in Figure 9.

**Figure 10:**
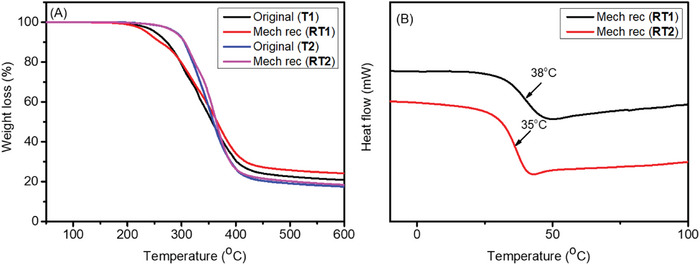
A) TGA and B) DSC curves mechanically reprocessed thermosets **1 (RT1)** and thermoset 2 **(RT2)**.

The mechanically reprocessed recycled thermosets (**RT1** and **RT2)** were characterized again, by using TGA and DSC as shown in **Figure** **10** and Table S2, Supporting Information. The thermal stability as investigated by TGA of recycled thermosets was very similar to the thermal stability of the original once compression molded thermosets. The glass transition temperatures (*T*_g_) of the recycled thermosets (**RT1** and **RT2)** were observed at 38 and 35 °C, respectively. These values are slightly higher, but in same range than those of the original thermosets (**T1** and **T2)**.

## 3 Conclusions

Two extended vanillin‐based imine monomers were successfully synthesized. The linear monomer 1 (**M1**) had two alcohol end groups and one imine bond, while the branched monomer 2 (**M2**) had three alcohol end‐groups and three imine‐groups. These monomers were further reacted with trimethylolpropane triglycidyl ether to produce thermoset 1 (**T1**) and 2 (**T2**). The structure of the monomers and thermosets was confirmed by NMR and FT‐IR spectroscopic techniques. Both thermosets exhibited good material properties and solvent resistance in common organic solvents. Finally, they were thermally reprocessable through compression molding with good recovery of the mechanical properties and they could be completely chemically recycled to water‐soluble aldehydes and amines by imine hydrolysis in acidic medium at room temperature. The developed thermosets, thus, show promise as future thermoset materials fitting the circular economy model.

## 4 Experimental Section

### Materials

Vanillin (VL) (99%), ethylene carbonate (EC) (98%), potassium carbonate (K_2_CO_3_, 99%), sodium sulphate (Na_2_SO_4_), dibutyltin oxide (DBTO) (>98%), 2‐aminoethanol (>99%), trimethylolpropane tris [poly (propylene glycol), amine terminated] ether (Jefm) (average Mn of 440 g mol^−1^), trimethylolpropane triglycidyl ether (TMPTGE), 1,2‐dimethylimidazole, and dibutyltin dilaurate (DBTDL) (>95%) were purchased from Sigma‐Aldrich. *N,N*‐dimethylformamide (DMF, ACS, Reag. Ph. Eur.), ethyl acetate (EtOAc, ACS, Reag. Ph. Eur.), and ethanol (EtOH) were purchased from VWR Chemicals. All chemicals and reagents were used as received.

### Synthesis of Extended Vanillin (ExtV, 3)

Vanillin (10.00 g, 0.066 mol), EC (6.36 g, 0.072 mol), K_2_CO_3_ (10.90 g, 0.079 mol), and 50 mL of DMF were added to a 250 round‐bottom flask with a magnetic stir bar. The solution temperature was kept at 110 °C for 12 h under nitrogen atmosphere. The completion of the reaction was monitored by TLC. After the reaction, the product was extracted using EtOAc with water (1:5), and then the organic layer was washed twice with water. Subsequently, the solution was dried with Na_2_SO_4_ and concentrated by using rotavapor. The product was recrystallized by using hot water and the collected product was thoroughly dried in a vacuum oven at 60 °C for 12 h.

White solid; yield 60%; ^1^H NMR (400.13 MHz, DMSO‐ d6): δ ppm 9.84 (s, 1H, —CHO), 7.55 (d, 1H, Ar), 7.40 (s, 1H, Ar), 7.19 (d, 1H, Ar), 4.93 (t, 1H, benzylic —OH), 4.10 (t, 2H, OCH_2_CH_2_OH), 3.84 (s, 3H, —OCH_3_) 3.76 (m, 2H, —OCH_2_CH_2_OH). ^13^C NMR (100.61 MHz, DMSO‐d6): δ ppm 191.54, 153.75, 149.28, 129.62, 126.21, 112.13, 109.61, 70.49, 59.43, and 55.53.^[^ ^16^, ^39^ ^]^

### General Procedure for the Synthesis of Schiff‐Base Monomers

ExtV (6.4 g, 0.033 mol) was dissolved in 20 mL of EtOH, and the corresponding amine compound (e.g., 2‐amino ethanol (1.98 g, 0.033 mol) or Jefm (5.00 g, 0.011 mol)) was added dropwise. The solution was stirred at room temperature under N_2_ atmosphere for 4 h. The completion of the reaction was monitored by TLC and the solvent was removed by using rota vapor and the product was dried with help of vacuum pump at 60 °C for 12 h, yielding the monomers 1 (5) and 2 (7), respectively.

### Monomer 1 (**M1**)

Yellow solid; yield 85%; mp 85 °C (DSC); ^1^H NMR (400.13 MHz, DMSO‐ d6): δ ppm 8.20 (s, 1H, imine), 7.37 (d, 1H, Ar), 7.19 (dd, 1H, Ar), 7.01 (d, 1H, Ar), 4.88 (t, 1H, —ArCH_2_CH_2_OH), 4.59 (t, 1H, —NCH_2_CH_2_OH), 4.01 (t, 2H, imine —CH_2_), 3.79 (s, 3H, —OCH_3_), 3.64 (t, 2H, imine —CH_2_CH_2_), and 3.59–3.63 (m, 4H, —OCH_2_CH_2_—). ^13^C NMR (100.61 MHz, DMSO‐d6): δ ppm 161.19, 150.35, 149.02, 129.24, 122.52, 112.27, 109.19, 70.15, 63.21, 60.89, 59.51, and 55.33. FTIR ν (cm^−1^): 3551, 3160, 2838, 1633, 1573, 1505, 1259, 1132, 1022, 912, 792, and 750.

### Monomer 2 (**M2**)

Slightly orange viscous liquid; yield 80%; ^1^H NMR (400.13 MHz, DMSO‐ d6): δ ppm 8.20 (s, 3H, imine), 7.33 (s, 3H, Ar), 7.17 (d, 3H, Ar), 6.99 (d, 3H, Ar), 4.88 (t, 3H, benzylic —OH), 3.99 (broad s, 6H, Ar—OCH_2_—), 3.77 (broad s, 9H, —OCH_3_), 3.74 (broad s, 6H, Ar—OCH_2_CH_2_—), and 3.51–0.97 (m, Jefm peaks). ^13^C NMR (100.61 MHz, DMSO‐d6): δ ppm 159.89, 150.81, 149.43, 129.62, 122.94, 112.55, 109.65, 76.15, 75.29, 74.71, 73.76, 70.57, 65.69, 64.94, 64.82, 59.91, 56.50, 55.71, 46.86, 46.70, 43.40, 20.34, 19.54, 19.47, 19.00, 17.68, and 7.94. FT‐IR ν (cm^−1^): 3356, 2871, 1641, 1589, 1513, 1444, 1255, 1088, 1012, 906, 792, and 750.

### Synthesis of Thermoset 1 (**T1**)

Monomer 1 **(M1)** (1 g, 0.0049 mol) and TMPTGE **(5)** (1.26 g, 0.0033 mol) with catalytic amount of 1,2‐dimethylimidazole (4 mg, 0.041 mmol) was added in a vial and kept at 70 °C under stirring for 15 min. When the solution became homogeneous, it was transferred to a teflon mold with the dimensions of 0.5 cm width and 8.0 cm length that was previously heated to 140 °C. After 4 h, the teflon mold was removed from the oven yielding the corresponding flexible thermoset 1.

FTIR ν (cm^−1^): 3381, 2872, 1590, 1505, 1446, 1259, 1090, 1013, 895, and 759.

### Synthesis of Thermoset 2 **(T2)**

Same procedure was followed, the Jeffamine (6) (1 g, 0,0010 mol) and TMPTGE (5) (0.3 g, 0.0010 mol) with a catalytic amount of 1,2‐dimethylimidazole (1 mg 0.010 mmol) were closed in a vial and kept at 70 °C under stirring for 15 min. When the solution became homogeneous it was transferred to a teflon mold that was previously heated at 140 °C. After 4 h, the teflon mold was removed from the oven, and the reaction was stopped yielding the corresponding flexible thermoset 2.

FTIR ν (cm^−1^): 3401, 2863, 1641, 1581, 1520, 1452, 1263, 1081, 1020, 921, 792, and 739.

### Processing and Reprocessing of Films by Compression Molding

The samples recovered from Teflon mold were cut into small pieces of about 5 mm × 5 mm each. The pieces were put in a mold placed inside a hot press and maintained for 30 min at 180 °C, with a pressure of 200 kN to obtain the virgin thermoset 1 and 2 films (**T1** and **T2**). The same procedure with cutting of films, followed by hot press was followed to obtain the reprocessed recycled thermosets (**RT1** and **RT2**).

### Characterizations

Attenuated total reflectance Fourier transform infrared spectroscopy (ATR‐FTIR): The precursors and the resins were analyzed with a PerkinElmer 2000 spectrophotometer equipped with an attenuated total reflection (ATR) setup. The analysis was made in a range of 400–4000 cm^−1^.

### Nuclear Magnetic Resonance Spectroscopy

The starting materials and monomers were analyzed using an Avance 400 spectrometer at 298 K. The samples were prepared by dissolving them in an NMR tube with deuterated DMSO, or D_2_O. The chemical shifts were reported as δ values (ppm). The residual DMSO‐d6 (2.50 and 39.52 ppm) and D_2_O (4.79 ppm) were taken as a reference. The proton and carbon frequencies were 400.13 and 100.61 MHz, respectively.

### Differential Scanning Calorimetry

The precursors and resins were analyzed using a Mettler Toledo DSC 1 instrument. The samples were studied with a heating rate of 10 °C min^−1^ under nitrogen flow with a purge rate of 50 mL min^−1^. The sequence consisted of a heating ramp from −30 to 200 °C, followed by a cooling ramp to 0 °C and finally a heating ramp to 200 °C. The glass transition temperature (*T*_g_) was determined from the second heating cycle. DSC data were analyzed by Mettler Toledo STARe v. 15.00 software.

### Thermogravimetric Analysis

TGA analysis was performed using a Mettler Toledo TGA 1 instrument at a heating rate of 10 °C min^−1^ under nitrogen atmosphere with a purge rate of 50 mL min^−1^ at temperature range 50–600°C. Before TGA analysis the samples were first heated to 120 °C and kept there isothermally for 5 min, to remove any solvent residue. TGA data were analyzed by Mettler Toledo STARe v. 15.00 software.

### Tensile Testing

Sample size of hot pressed thermosets (**T1** and **T2**) and recycled thermosets (**RT1** and **RT2**) was performed by Instron 5944 universal testing machine. The sample dimensions were 25 mm × 5.0 mm × 0.5 mm, similar dimensions were followed for the reprocessed samples. All the samples were conditioned at 22 °C and 40% relative humidity for 2 days before testing.

### Solvent Resistance

Samples with an approximate weight of 50 mg were immersed in 10 mL of different solvents (acetone, THF, DMF, DCM, 1 m NaOH, EtOH, DMSO) at room temperature for 2 days. Remaining material % was calculated as

(1) $$\begin{array}{c} \text{Remaining}\;\text{material}\%\;=\;M_{d}/M_{o}x100 \end{array}$$

where (*M*_o_) denotes original weight and (*M*_d_) is the dry weight after solvent exposure.

### Chemical Recycling

50 mg of each thermoset was placed in a vial filled with 2 mL of hydrochloric acid (0.1 m HCl) for 2 h. The solvents were evaporated using vacuum oven, the samples were weighted and analyzed by NMR after dissolving in D_2_O.

## Conflict of Interest

The authors declare no conflict of interest.

## Supporting information

Supporting Information

## Data Availability

The data that support the findings of this study are available from the corresponding author upon reasonable request.

## References

[1] J. M. Raquez , M. Deléglise , M. F. Lacrampe , P. Krawczak , Prog. Polym. Sci. 2010, 35, 487.

[2] T. Kaiser , Prog. Polym. Sci. 1989, 14, 373.

[3] A. Shiota , C. K. Ober , Prog. Polym. Sci. 1997, 22, 975.

[4] J. Zhang , J. Huang , G. Zhu , X. Yu , J. Cheng , Z. Liu , Y. Hu , Q. Shang , C. Liu , L. Hu , Y. Zhou , Green Chem. 2021, 23, 5875.

[5] M. Hakkarainen , Y. Xu , K. Odelius , ACS Sustainable Chem. Eng. 2020, 8, 17272.

[6] D. Montarnal , M. Capelot , F. Tournilhac , L. Leibler , Science 2011, 334, 965. 22096195 10.1126/science.1212648

[7] B. Krishnakumar , A. Pucci , P. P. Wadgaonkar , I. Kumar , W. H. Binder , S. Rana , Chem. Eng. J. 2022, 433, 133261.

[8] J. Han , T. Liu , C. Hao , S. Zhang , B. Guo , J. Zhang , Macromolecules 2018, 51, 6789.

[9] M. Capelot , D. Montarnal , F. Tournilhac , L. Leibler , J. Am. Chem. Soc. 2012, 134, 7664. 22537278 10.1021/ja302894k

[10] J. P. Brutman , P. A. Delgado , M. A. Hillmyer , ACS Macro Lett. 2014, 3, 607. 35590755 10.1021/mz500269w

[11] Z. Q. Lei , H. P. Xiang , Y. J. Yuan , M. Z. Rong , M. Q. Zhang , Chem. Mater. 2014, 26, 2038.

[12] A. R. De Luzuriaga , R. Martin , N. Markaide , A. Rekondo , G. Cabañero , J. Rodríguez , I. Odriozola , Mater. Horiz. 2016, 3, 241.

[13] W. Denissen , G. Rivero , R. Nicolaÿ , L. Leibler , J. M. Winne , F. E. Du Prez , Adv. Funct. Mater. 2015, 25, 2451.

[14] S. Engelen , A. A. Wróblewska , K. De Bruycker , V. Ladmiral , S. Caillol , F. Du Prez , Polym. Chem. 2022, 13, 2665.

[15] A. Liguori , M. Hakkarainen , Macromol. Rapid Commun. 2022, 43, 2100816. 10.1002/marc.202100816 35080074

[16] A. Liguori , S. Subramaniyan , J. G. Yao , M. Hakkarainen , Eur. Polym. J. 2022, 178, 111489.

[17] N. Jarach , H. Dodiuk , S. Kenig , N. Naveh , J. Appl. Polym. Sci. 2022, 139, e52353.

[18] X. L. Zhao , Y. Y. Liu , Y. Weng , Y. D. Li , J. B. Zeng , ACS Sustainable Chem. Eng. 2020, 8, 15020.

[19] P. Li , S. Ma , J. Dai , X. Liu , Y. Jiang , S. Wang , J. Wei , J. Chen , J. Zhu , ACS Sustainable Chem. Eng. 2017, 5, 1228.

[20] E. D. Hernandez , A. W. Bassett , J. M. Sadler , J. J. La Scala , J. F. Stanzione , ACS Sustainable Chem. Eng. 2016, 4, 4328.

[21] S. Wang , S. Ma , C. Xu , Y. Liu , J. Dai , Z. Wang , X. Liu , J. Chen , X. Shen , J. Wei , J. Zhu , Macromolecules 2017, 50, 1892.

[22] S. Ma , D. C. Webster , F. Jabeen , Macromolecules 2016, 49, 3780.

[23] S. Ma , D. C. Webster , Macromolecules 2015, 48, 7127.

[24] R. Auvergne , S. Caillol , G. David , B. Boutevin , J. P. Pascault , Chem. Rev. 2014, 114, 1082. 24125074 10.1021/cr3001274

[25] J. R. Rochester , Reprod. Toxicol. 2013, 42, 132. 23994667 10.1016/j.reprotox.2013.08.008

[26] W. Xie , S. Huang , S. Liu , J. Zhao , Chem. Eng. J. 2021, 404, 126598.

[27] D. Zhao , J. Wang , X. Wang , Y. Wang , Chem. Eng. J. 2018, 344, 419.

[28] A. Yang , C. Deng , H. Chen , Y. Wei , Y. Wang , Polym. Degrad. Stab. 2017, 144, 70.

[29] L. Jiang , Y. Tian , J. Cheng , J. Zhang , Polym. Chem. 2021, 12, 6527.

[30] A. W. Bassett , A. E. Honnig , C. M. Breyta , I. C. Dunn , J. J. La Scala , J. F. Stanzione , ACS Sustainable Chem. Eng. 2020, 8, 5626.

[31] X. Xu , S. Ma , J. Wu , J. Yang , B. Wang , S. Wang , Q. Li , J. Feng , S. You , J. Zhu , J. Mater. Chem. A 2019, 7, 15420.

[32] Y. Sun , D. Sheng , H. Wu , X. Tian , H. Xie , B. Shi , X. Liu , Y. Yang , Polymer 2021, 233, 124208.

[33] G. Li , X. Zhang , J. Huang , T. Li , S. Yang , Y. Wang , J. Jiang , B. Xia , M. Chen , W. Dong , Chem. Eng. J. 2022, 435, 134766.

[34] Y. Liu , Z. Yu , G. Lu , W. Chen , Z. Ye , Y. He , Z. Tang , J. Zhu , Chem. Eng. J. 2023, 451, 139053.

[35] K. Liang , G. Zhang , J. Zhao , L. Shi , J. Cheng , J. Zhang , ACS Sustainable Chem. Eng. 2021, 9, 5673.

[36] P. Wu , X. Wang , R. Shi , H. Cheng , F. Zhao , Green Chem. 2022, 24, 1561.

[37] A. S. Mora , R. Tayouo , B. Boutevin , G. David , S. Caillol , Molecules 2019, 24, 3285. 31505884 10.3390/molecules24183285 PMC6766844

[38] S. Zhao , M. M. Abu‐omar , Macromolecules 2018, 51, 9816.

[39] X. Li , X. Wang , S. Subramaniyan , Y. Liu , J. Rao , B. Zhang , Biomacromolecules 2022, 23, 150. 34932316 10.1021/acs.biomac.1c01186 PMC8753607

