# Supplementary material for: Vanillin‐Derived Thermally Reprocessable and Chemically Recyclable Schiff‐Base Epoxy Thermosets
Source: Glob Chall. 2023 Feb 7;7(4):2200234. doi: 10.1002/gch2.202200234 (PMC10069320; doi:10.1002/gch2.202200234)
Supplement: Supplementary file 1 — Supporting Information [file GCH2-7-2200234-s001.pdf]

Global
Challenges
Open Access

Supporting Information

for *Global Challenges,* DOI: 10.1002/gch2.202200234

Vanillin-Derived Thermally Reprocessable and Chemically Recyclable Schiff-Base Epoxy Thermosets

*Sathiyaraj Subramaniyan, Matteo Bergoglio, Marco Sangermano, and Minna Hakkarainen**

Supporting Information

Vanillin-derived thermally reprocessable and chemically recyclable Schiff-base epoxy thermosets

Sathiyaraj Subramaniyan[1,2], Matteo Bergoglio[1,3], Marco Sangermano[3] and Minna Hakkarainen[1,2]*

[1] KTH Royal Institute of Technology, Department of Fibre and Polymer Technology, Teknikringen 58, 100 44 Stockholm, Sweden
[2] KTH Royal Institute of Technology, Wallenberg Wood Science Center (WWSC), Teknikringen 58, 100 44 Stockholm, Sweden
[3]Politecnico di Torino, Department of Applied Science and Technology, C.so Duca degli Abruzzi 24, 10129 Torino, Italy
*Email: minna@kth.se

This supporting information contains 4 Figures and 2 Tables on 4 pages.

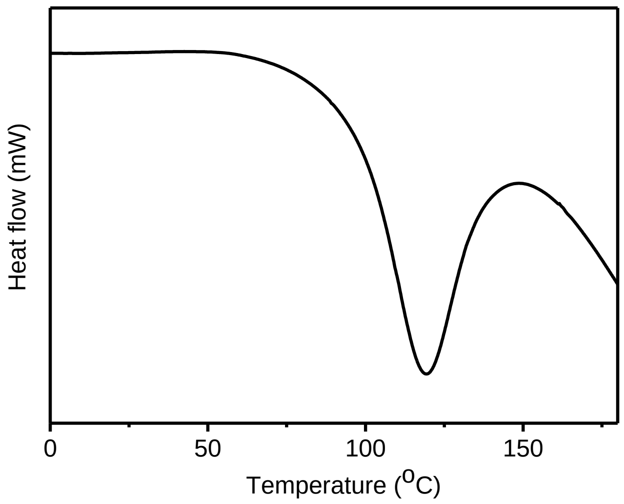

Figure S1. DSC heating scans for non-isothermal curing of monomer (**M1**) and TMPTGE in the presence of DMeIm at a heating rate of 10 °C min^{-1}.

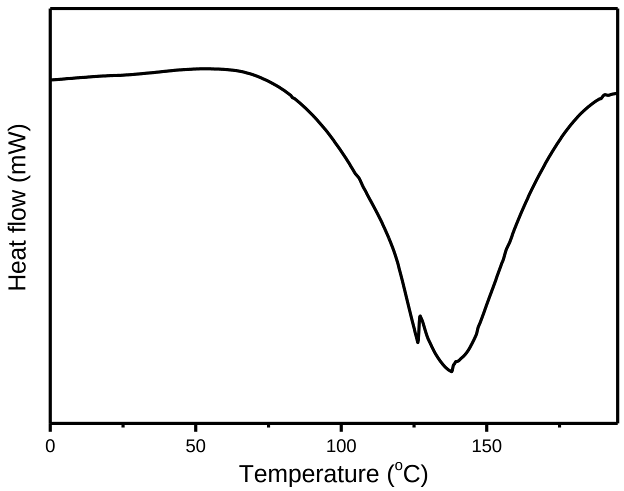

Figure S2. DSC heating scans for non-isothermal curing of monomer (**M2**) and TMPTGE in the presence of DMeIm at a heating rate of 10 °C min^{-1}.

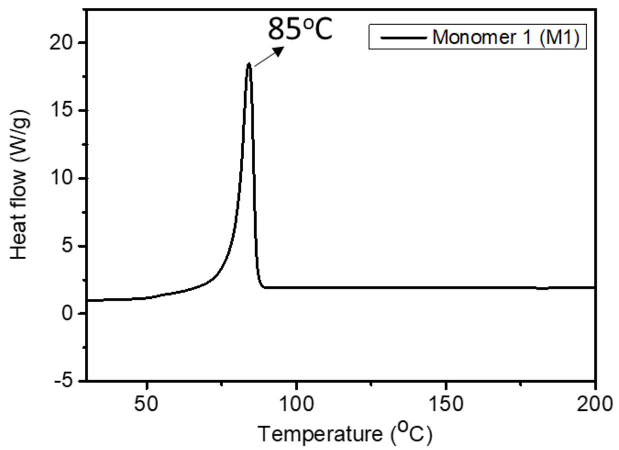

Figure S3. DSC curve of monomer (**M1**)

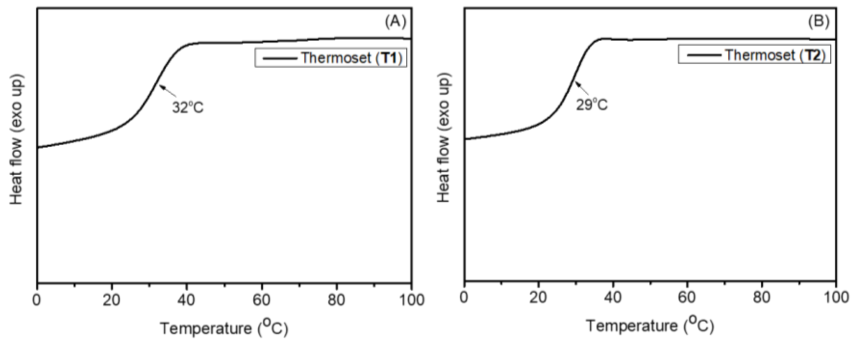

Figure S4. DSC curves of (A) thermoset 1 **(T1)** and (B) thermoset 2 **(T2)**.

Table S1. Solvent resistance evaluation performed on thermosets **T1** and **T2** immersed in different solvents for 48 h.

solvents	Remaining material %	
	Thermoset (**T1**)	Thermoset (**T2**)
Acetone (Ace)	89 ± 1	79 ± 4
Tetrahydrofuran (THF)	89 ± 2	77 ± 3
N,N-Dimethylformamide (DMF)	84 ± 4	74 ± 1
1,2-Dicholoromethane (DCM)	81 ± 3	76 ± 3
Sodium hydroxide 1M (NaOH)	99 ± 1	99 ± 1
Ethanol (EtOH)	94 ± 5	79 ± 4
Dimethylsulphoxide (DMSO)	88 ± 4	78 ± 1

Table S2. Tensile properties of thermosets **T1** and **T2,** and reprocessed thermosets (**RT1** and **RT2**).

Samples	Elastic modulus (MPa)	Tensile stress at break (MPa)	Extension at break (%)
Thermoset 1 **(T1)**	26.6 ± 2.0	13.6 ± 4.5	64.5 ± 10.6
Mechanically Recycled Thermoset 1 **(RT1)**	39.3 ± 3.8	10.2 ± 3.1	49.0 ± 5.4
Thermoset 2 **(V2)**	34.9 ± 5.3	16.8 ± 6.7	86.2 ± 16.0
Mechanically Recycled Thermoset 2 **(RT2)**	42.3 ± 3.0	15.6 ± 5.1	59.0 ± 19.5
